# The influence of input and output modality on following instructions in working memory

**DOI:** 10.1038/srep17657

**Published:** 2015-12-04

**Authors:** Tian-xiao Yang, Richard J. Allen, Qi-jing Yu, Raymond C. K. Chan

**Affiliations:** 1 Neuropsychology and Applied Cognitive Neuroscience Laboratory, Key Laboratory of Mental Health, Institute of Psychology, Chinese Academy of Sciences, Beijing, China; 2 School of Psychology, University of Leeds, UK; 3 Department of Psychology, Wayne State University, USA

## Abstract

Following instructions is an important component of learning and has been shown to rely on working memory. This study examined the ability to follow instructions within working memory under varying input and output modalities. In Experiment 1, participants heard, read, or viewed demonstration of short sequences of instructions, and recalled either by oral repetition or physical enactment. There was a significant main effect of encoding, showing superior recall performance when instructions were demonstrated relative to spoken or written presentation. Experiment 2 examined whether recall is further improved when instructions are presented both in spoken and demonstrated form, relative to single modality presentation. The advantage for demonstration over spoken instructions was replicated, and dual input was superior to spoken instructions. However, dual input did not bring extra benefit compared to demonstration of instructions. We also observed a significant enacted-retrieval recall advantage. These findings suggest effects of both input and output modalities on the ability to remember and follow instructions in working memory. Outcomes substantially inform the underexplored but important new area of action-based working memory and its links to embodied cognition, with implications for pedagogic practice.

Performing actions to command is a capacity that plays a key role in supporting everyday activities, e.g., rehearsing an instructor’s commands when learning to drive, cooking new dishes following a recipe, or imitating a leading dancer’s new movements. Instructions can vary in their input and output modalities, being delivered by oral commands, written directions, or demonstration. Response format can also vary, as instructions may be recalled through oral repetition or physical enactment. Little is known about the influence of presentation and response formats on the ability to follow instructions within working memory; exploring this is the primary goal of the current study, with the intention of identifying implications for memory function and for the optimal methods of presenting and encoding instructions in practical settings. To our knowledge, this is the first study to directly examine verbal recall vs. physical enactment of instructions in a working memory task following demonstration, written, and spoken presentation.

A body of earlier research exists investigating the enactment effect in memory for action events^1^,^2^,^3^,^4^,^5^. In a typical task setting, participants were required to memorize verbal lists of actions, followed by later verbal recall or recognition tasks. Having the participant or ‘subject’ (Subject Performed Task, SPT) or the experimenter (Experimenter Performed Task, EPT) performing the actions during encoding improved recall^6^. Compared to EPT, the SPT effect was larger and more stable^5^, which may reflect different encoding processes, with EPT possibly providing visual-imaginal encoding while SPT benefits from motor encoding^7^ and involvement of self^8^. It should be noted that the paradigm used in these earlier studies differed from the following instructions task presently under examination. First, the list of actions used in these studies usually contained more than 10 actions, thus largely exceeding the capacity of working memory^9^ and drawing on long-term memory even when immediate recall was required. Second, unlike the following instructions paradigm, these studies often used free recall without requiring serial coding of actions, which may reduce the contribution of working memory to serial coding^10^. Finally, they were typically restricted to assessing retention with verbal recall or recognition, rather than via physical enactment.

Indeed, following instruction is a complex cognitive process that involves multiple cognitive functions and in particular, working memory, a limited capacity system that enables us to hold and manipulate information for a brief period of time^11^,^12^,^13^. Research has shown that a child’s ability to follow teacher’s oral commands in the classroom is closely associated with working memory capacity^14^,^15^. Moreover, experiments using dual-task methodology have indicated that remembering instructions is cognitively demanding and relies on phonological and visuospatial working memory and attentional control^16^.

Given that following instructions is a common requirement in the classroom environment, and that impairments in this ability may contribute to broader academic difficulties encountered by children with poor working memory^17^, it is important to identify optimal ways in which instructions should be provided. A key factor that remains underexplored in this context is input modality. In the few studies in a working memory context to date, SPT or EPT improved memory recall relative to pure verbal encoding^18^,^19^. However, previous work on verbal instructions focused on spoken or written presentation^14^,^15^,^16^,^18^,^19^,^20^, with little exploration of memory for visually demonstrated instructions. Wood^21^ has argued for a limited capacity system for storing observed actions that is independent of memory for visual and spatial information. Demonstration may therefore provide an additional form of coding to support working memory performance. More generally, imitating the actions of others starts early in life, usually before mastering language^22^,^23^ and may occur automatically via direct mapping, possibly reflecting ‘resonance’ in the motor areas of the brain in response to observed actions that may occur even when actions are not executed^24^. Therefore, in the context of remembering series of actions, an advantage in recall may emerge when instructions are visually demonstrated rather than verbal in nature. This key question was examined through a comparison of single modality presentation formats (spoken, written, or demonstration) in Experiment 1, and comparing single- with dual-modality (i.e. spoken or demonstration only vs. spoken plus demonstration) in Experiment 2.

In addition to input modality, this study also explored the influence of output format. An important phenomenon noted in the literature is the retrieval-enactment advantage, i.e., the finding that enacted recall is superior to verbal repetition of instructions^15^,^16^,^19^. Similarly, Koriat *et al.*^25^ found that memory for written instructions depended more on expected than actual mode of report, suggesting that enactment benefits arise in part during encoding rather than retrieval. Active action planning may facilitate association of movements with environmental cues within a temporal framework^26^ and help form an integrative representation resulting in improved enacted recall^19^,^27^,^28^. This may not develop to the same extent when verbal repetition is required, as instructions can be maintained via simple rehearsal, though less accurately than when planning for enactment. However, while previous studies have observed this advantage using either spoken or written instructions^16^,^29^, whether it also emerges following demonstrated instructions remains to be investigated. Visual demonstration may result in representational forms that are more appropriate for subsequent physical enactment rather than verbal repetition, thus leading to an increased retrieval-enactment effect. Alternatively, as action planning may already be activated during encoding for anticipated enacted recall, demonstration benefits may be reduced in this condition, reflecting a common representational domain for planning and perceiving demonstrated actions^30^.

The current study therefore reports two experiments manipulating input and output modality of instructions. In both experiments, participants were required to recall instructions either by oral repetition or enactment. In Experiment 1, input modalities involved spoken, written, and demonstrated presentation. In Experiment 2, spoken, demonstration and dual (simultaneous demonstration and spoken instructions) input modalities were examined.

## Experiment 1

There were two predictions in this first experiment. First, there would be an input modality effect, emerging as superior performance following demonstration compared to verbal-based instruction (both spoken and written instructions). Second, there would be an output modality effect, with an advantage of recalling by enactment relative to oral repetition.

With regard to spoken and written instructions, each has associated costs and benefits. Spoken instructions permit the simultaneous encoding of verbal instruction alongside visual scanning of information within the environment, possibly facilitating performance^31^. In addition, spoken instructions may benefit from the enhanced recall of final items when presented auditorily rather than visually^32^. In contrast, written presentation allows participants to encode and selectively rehearse instructions at their own pace, as well as providing opportunities for applying mnemonic strategies, such as visual coding of words and selectively rehearsing difficult action phrases. Therefore, no a priori prediction regarding relative performance levels of these two types of instructions was made.

### Results

**Figure 1:**
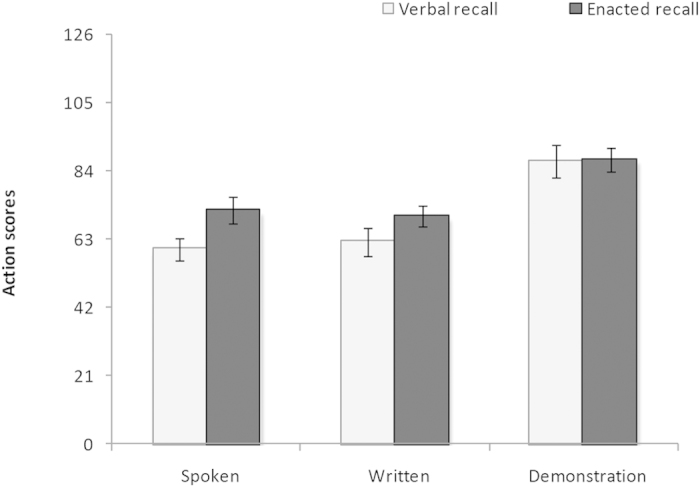
Mean total action scores (with error bars showing standard error) as a function of input and output modality in Experiment 1.

Descriptive results for the action scores for each condition are displayed in Fig. 1. An action was scored as correct only when the combination of movement, colour, shape was correct and the action was in the correct serial position. The possible scores ranged from 0 to 126. In addition, descriptive results of span scores are provided in Table S1 in the online supplementary materials. As action scores provided a larger score range compared to span scores, the following ANOVA analyses focused on this dependent variable.

A 3× 2 (Modality × Recall Type) ANOVA showed a significant main effect of input modality, *F*_(2, 68)_ = 22.31, *p* < 0.001, *η*_*p*_^*2*^ = 0.40. There was a marginal nonsignificant main effect of output modality, with a trend toward superior performance of enactment recall relative to verbal recall, *F*_(1,34)_ = 3.46, *p* = 0.072, *η*_*p*_^*2*^ = 0.09. There was no significant interaction between input and output modality overall, *F*_(2, 68)_ = 1.18, *p* = 0.313, *η*_*p*_^2^ = 0.03. A series of 2 × 2 ANOVAs were then conducted to test specific effects of input modality. A 2 (Demonstration-Spoken) × 2 (Recall Type) ANOVA revealed significantly higher performances in demonstration compared to spoken condition, *F*_(1,34)_ = 35.11, *p* < 0.001, *η*_*p*_^*2*^ = 0.51, and no significant main effect of recall type, *F*_(1,34)_ = 2.00, *p* = 0.166, *η*_*p*_^*2*^ = 0.06, nor interaction, *F*_(1,34)_ = 2.39, *p* = 0.132, *η*_*p*_^*2*^ = 0.07. A 2 (Demonstration-Written) × 2 (Recall Type) ANOVA revealed significantly higher performances in demonstration compared to written condition, *F*_(1,34)_ = 30.40, *p* < 0.001, *η*_*p*_^*2*^ = 0.47, and no significant main effect of recall type, *F*_(1,34)_ = 0.96, *p* = 0.333, *η*_*p*_^*2*^ = 0.03, nor interaction, *F*_(1,34)_ = 0.87, *p* = 0.357, *η*_*p*_^*2*^ = 0.03. A 2 (Spoken-Written) × 2 (Recall Type) ANOVA revealed similar performances in written and spoken condition, *F*_(1,34)_ = 0.01, *p* = 0.312, *η*_*p*_^*2*^ = 0.94, and a significant main effect of recall type, *F*_(1,34)_ = 6.40, *p* = 0.016, *η*_*p*_^*2*^ = 0.16, but no interaction, *F*_(1,34)_ = 0.31, *p* = 0.580, *η*_*p*_^*2*^ = 0.01.

### Discussion

Consistent with our hypothesis, we observed a benefit of demonstration relative to spoken and written instructions in working memory. Demonstration allows perceiving and later imitating others’ actions, which may trigger direct mapping in motor areas and automatic formation of an integrative representation^24^,^33^. Previous work has indicated improved free recall of multiple actions performed by others compared to simple verbal learning^5^, suggesting a demonstration benefit in long-term memory. The current study is novel in extending this to working memory. In contrast, spoken and written presentation did not significantly differ in resulting performance levels. These findings suggest an intrinsic difference between demonstration and verbal instructions, with the former primarily utilizing visuospatial cues whereas the latter relies mainly on verbal coding, which may be less optimal for processing action-based instructional content.

There was an overall marginal advantage for enactment recall relative to oral repetition of instructions, which was generally consistent with our second hypothesis. Further examination revealed an enacted recall advantage in verbal (spoken and written) instructions in contrast to an absence of this effect when demonstration was involved. This pattern lends some support to the prediction that the enactment advantage in spoken instructions would be reduced or even eliminated when instructions are demonstrated given the common representation of action perception and planning^34^. However, as the interaction between input and output modality did not reach significance in this experiment, caution should be taken when interpreting this outcome.

## Experiment 2

There were two aims in this experiment. First, the novel finding of the demonstration advantage in Experiment 1 requires replication. Second, we explored whether a benefit for multiple modalities emerges in contrast to single modality presentation. Studies in multimedia educational domains have consistently shown the benefit of presenting information through different modalities during a learning scenario when working memory load is high^35^. Therefore, it is possible that presenting spoken instructions along with demonstrated actions can facilitate memory performance relative to single modality presentation. Within a working memory context, Wojcik, *et al.*^18^ observed superior action performance in children with autism spectrum disorder (ASD) and age-matched controls following demonstration and verbal instruction, relative to verbal-only presentation, suggesting that demonstration facilitates following of instructions. However, no previous studies have contrasted dual-modality with demonstration-only presentation, or examined these questions using both verbal and enacted recall. Single and dual input conditions were therefore included, with the latter involving simultaneous spoken instructions paced with demonstrated actions.

### Results

**Figure 2:**
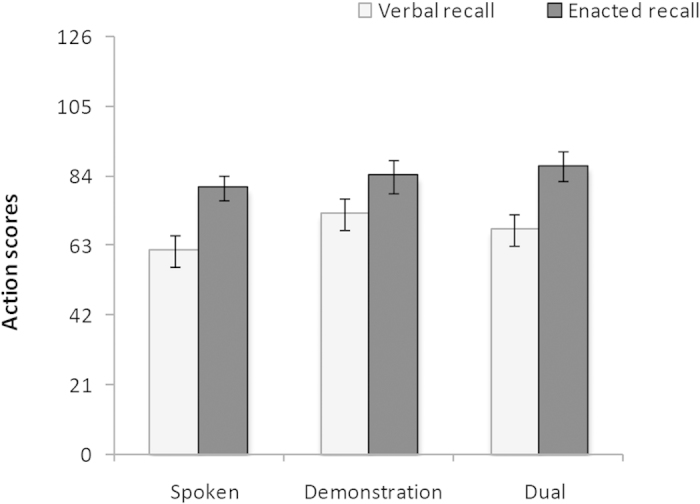
Mean total action scores (with error bars showing standard error) as a function of input and output modality in Experiment 2.

Descriptive results for the action scores for each condition are displayed in Fig. 2. A 3 × 2 (Modality × Recall Type) ANOVA on action scores showed a significant main effect of output modality, with enactment recall superior to verbal recall, *F*_(1,34)_ = 10.62, *p* = 0.003, *η*_*p*_^*2*^ = 0.24. There was a marginal nonsignificant main effect of input modality, *F*_(2,68)_ = 2.85, *p* = 0.065, *η*_*p*_^*2*^ = 0.08, and the interaction between input and output modality was not significant, *F*_(2, 68)_ = 0.88, *p* = 0.418, *η*_*p*_^*2*^ = 0.03. A series of 2 × 2 ANOVAs were conducted to test the specific effects of input modality. A 2 (Demonstration-Spoken) × 2 (Recall Type) ANOVA replicated the demonstration advantage from Experiment 1, showing significantly higher performance in demonstration compared to spoken condition, *F*_(1,34)_ = 4.86, *p* = 0.034, *η*_*p*_^*2*^ = 0.13, and a significant main effect of recall type, *F*_(1,34)_ = 7.46, *p* = 0.010, *η*_*p*_^*2*^ = 0.18, but no significant interaction, *F*_(1,34)_ = 1.31, *p* = 0.261, *η*_*p*_^*2*^ = 0.04. A 2 (Dual-Demonstration) × 2 (Recall Type) ANOVA revealed no significant difference between dual and demonstration conditions, *F*_(1,34)_ = 0.06, *p* = 0.803, *η*_*p*_^*2*^ = 0.02, and an enactment recall advantage, *F*_(1,34)_ = 7.84, *p* = 0.008, *η*_*p*_^*2*^ = 0.19, but no interaction, *F*_(1,34)_ = 1.24, *p* = 0.273, *η*_*p*_^*2*^ = 0.04. Finally, a 2 (Dual-Spoken) × 2 (Recall Type) ANOVA revealed a marginally superior performance in the dual condition compared to spoken condition, *F*_(1,34)_ = 4.02, *p* = 0.053, *η*_*p*_^*2*^ = 0.11, and a significant enactment recall advantage, *F*_(1,34)_ = 13.10, *p* = 0.001, *η*_*p*_^*2*^ = 0.28, but no interaction, *F*_(1,34)_ < 0.01, *p* = 0.973, *η*_*p*_^*2*^ < 0.01.

### Discussion

Consistent with the first hypothesis, memory performance in demonstration conditions was superior to that in spoken instructions, thus replicating Experiment 1 and suggesting the demonstration advantage to be a reliable effect. The second aim concerns the comparison of single versus dual modality input. Memory performance was similar in dual and demonstration condition, indicating that adding additional spoken instructions to demonstration did not bring extra benefit. In contrast, there was a marginal advantage for recall in the dual-modality condition relative to spoken instruction, suggesting additional benefit of perceiving actions while listening to the instructions^18^. These results all indicate a substantial advantage for encoding via demonstration; as long as this was involved during input, memory performance was enhanced.

There was also a clear retrieval-enacted recall advantage, which emerged following both spoken instruction and demonstration. Consistent with Experiment 1, there was no interaction between input (spoken vs. demonstration) and output modality, although a trend again emerged for a somewhat larger enactment recall benefit following spoken presentation (and a larger demonstration advantage for verbal recall). However, as this was not sufficiently robust to reach statistical significance, it should be treated with caution.

## Combining data of Experiment 1 and 2

In Experiment 1 and 2, there was a trend for a larger enactment recall advantage in spoken relative to demonstration conditions, and a larger benefit of demonstration over spoken instructions in verbal than enactment recall condition. To further clarify these effects using increased statistical power, we merged the data from spoken and demonstration conditions in Experiment 1 and 2. A 2 (Demonstration-Spoken) × 2 (Recall Type) ANOVA indicated significantly higher performances in demonstration compared to spoken condition (*F*_(1,70)_ = 31.36, *p* < 0.001, *η*_*p*_^*2*^ = 0.31), a significant enacted recall advantage (*F*_(1,70)_ = 9.12, *p* = 0.004, *η*_*p*_^*2*^ = 0.12); and a trend for an interaction between these factors (*F*_(1,70)_ = 3.35, *p* = 0.071, *η*_*p*_^*2*^ = 0.05). Further t-tests indicated a significant demonstration benefit (over spoken instructions) in both verbal (*t*_(35)_ = 5.06, *p* < 0.001, Cohen’s *d* = 1.01) and enactment recall condition (*t*_(35)_ = 2.78, *p* = 0.009, Cohen’s *d* = 0.57). The enactment recall advantage emerged following spoken instruction (*t*_(70)_ = 3.81, *p* < 0.001, Cohen’s *d* = 0.92), but not in the demonstration condition (*t*_(70)_ = 1.28, *p* = 0.205, Cohen’s *d* = 0.31).

## General Discussion

This study investigated the influence of input and output modality on following instructions in working memory. Both input and output modality significantly influenced memory performance for short sequences of instructions. In terms of input modality, demonstration has a clear advantage over verbal instructions (Experiments 1 and 2), indicating this to be a superior method of instruction provision. Furthermore, Experiment 2 demonstrated that adding visual demonstration to auditory instruction resulted in improved recall, in line with the principles of dual-coding facilitation^36^. This benefit was similar to the EPT effect observed in long-term memory for action events^5^ and extends it to the immediate serial recall of short action sequences, a task that places high demands on working memory^16^. However, this effect was not reciprocal, with recall following dual-modality presentation (i.e. verbal + demonstration) no better than demonstration-only. This would indicate multi-modality effects in the present paradigm to be non-additive and that spoken instruction is not informative for instruction recall above and beyond input gained from observing demonstration. These findings may also be consistent with the suggestion that action representations are robust and immune to rapid decay compared to sensory representation^37^. Future study should further investigate the nature of action representations from a working memory perspective, particularly given the proposed contribution of the episodic buffer within the multicomponent working memory model^12^,^13^,^38^. In particular, it would be useful to establish whether visual and spoken input channels are integrated and the extent to which this requires active processing^12^,^31^, or develops relatively automatically during observation and action planning^33^.

In terms of output modality, an enactment recall advantage was observed across the two experiments. This effect was relatively stable when spoken instructions were involved, replicating previous findings^15^,^16^,^19^,^29^, but was numerically smaller and less reliable in demonstration conditions. This pattern is generally consistent with the hypothesis of a common representation of action perception and action planning^34^, and with recent observations that the retrieval-enactment advantage is substantially reduced when instructions are enacted by the participant during encoding^19^; although as encoding by recall interaction outcomes did not reach the *p* < 0.05 criterion in the present experiments, the relationships between these factors should be treated with caution. Nevertheless, it appears that provided the task involves action or enactment (either through active action planning for later enactment recall, or action perception during encoding), enhanced memory performance can be observed compared to purely verbal encoding. Finally, it should be noted that, regardless of variations in relative size, large demonstration effects were observed for both verbal and enacted recall. The facilitatory forms of coding obtained through demonstration are therefore not limited to a particular response format.

In summary, this study has provided the first evidence that both input and output presentation modality impacts on the ability to follow instructions, and provides new insights into effects of demonstration at encoding and enactment at retrieval. While people appear capable of holding around four object-action chunks in working memory^9^, this can vary under different encoding and response conditions. These findings have implications for educators and designers, who may utilize the benefits of demonstration and enactment across different contexts. Furthermore, the instructional span task developed in this study provides a measurement of working memory capacity involving actions that can be used to test action-based working memory in various populations. Our work builds on and extends previous research on memory for action events^3^,^39^ by showing that the demonstration benefit also emerges in serial verbal and enactment recall of short action sequences, a paradigm that particularly emphasizes working memory. Our findings thus substantially inform the relatively underexplored but important new area of action-based working memory. Improvements in performance as a result of visual demonstration and enacted retrieval, together with recently observed benefits from enactment during encoding^19^ all represent the positive influence of including action-related processing in memorization. These findings in turn may correspond to the notion of embodied cognition by emphasizing the benefit of perceptual and bodily interactions with a three-dimensional world^40^ in which memory plays a key role in guiding actions. Future work should continue to investigate the cognitive and neural mechanisms that contribute to working memory for action and instruction, in order to better understand the nature of action-related processing.

## Methods

The two experiments were approved by the ethic committee of the Institute of Psychology, Chinese Academy of Sciences. The methods in the two experiments were carried out in accordance with the approved guidelines. Consent form was obtained from all participants.

### Experiment 1

#### Participants

Thirty-six native Mandarin Chinese speakers were recruited through phone appointment. There were 18 females and 18 males, aged from 18 to 25, with a mean age of 22.22 and 15.58 mean years of education.

#### Materials

The instructions contained series of actions carried out on a subset of 12 objects. There were five types of action phrases (touch, push, drag, spin, pick up… put it into…) and twelve items of coloured stationery, including six small objects (a yellow ruler, a blue ruler, a white eraser, a green eraser, a red pencil and a black pencil) and six containers (a yellow basket, a white basket, a blue folder, a green folder, a red bag and a black bag). The objects in each instructional sequence were selected randomly, with the constraint of no repetition of features (action phrases, colours, objects) for adjacent actions within the sequence. An example of a three-action sequence was “pick up white eraser, put it into yellow basket, touch green folder”. As a span procedure was used, instructions were organized into six blocks with actions increasing from one to six. Each block contained six instructional sentences (examples can be found as Supplementary material online). Three parallel instruction lists were created, and each list contained six blocks and 36 instructional sentences, which were recorded in three input formats (i.e., spoken, written and demonstration).

Spoken instructions were recorded by a Native Chinese female speaker at a moderate speed (approx. 350 ms per word), and presented to participants through speakers. For the written instructions condition, instructions were presented on a computer screen via the Eprime software. Demonstration of instructions was provided through video clips comprising series of hand movements upon objects. The durations of instructions were consistent across the three modalities, and varied with the number of actions. For sequences of 1 to 6 actions, the durations were 3, 5, 8, 11, 13 and 16 seconds respectively.

#### Design and Procedure

In a 3 × 2 mixed design, input modality was a within-subject variable, including spoken, written and demonstration conditions. Output modality was a between-subject variable, including verbal and enactment recall. The dependent variables were maximum span score and number of correct action-object pairs. A response was only scored as correct when produced in the correct serial position.

Each participant was first introduced to the experiment and then signed the consent form. Each participant was randomly assigned to one of the recall groups, and completed all presentation conditions. Three sets of instructional sequences were implemented in counterbalanced order for each participant, with each condition utilizing the same set an equal number of times.

**Figure 3:**
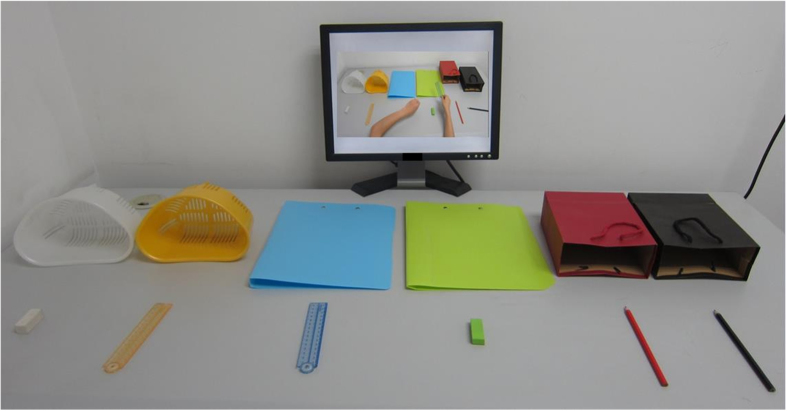
Illustration of task set-up during the demonstration condition.

Participants sat at a 150 cm × 70 cm × 75 cm desk, facing the objects and a computer monitor for displaying instructions (see Fig. 3). The experimenter sat at another desk 100 cm away from the participants, controlling the delivery of instructions. The experimenter first introduced the task, followed by a practice of object naming in the verbal-recall group or an operation exercise in the enactment group, in order to ensure that participants understood the instructions and the recall requirements. Participants were told that repeating the instructions aloud, and touching, operating or moving the objects during encoding were all forbidden.

In a typical spoken instruction trial, the experimenter first signalled the participants to get ready, and then triggered presentation through speakers. In the written instructions condition, each action-object pair was simultaneously presented in separate rows on the computer monitor, with the texts centred and a typical Chinese font of size 16. In the demonstration condition, participants viewed silent video clips of actions. In all conditions, a blank screen would appear at the end of the trial, indicating the recall phase. Based on recall condition, participants either repeated the instructions (verbal recall) or performed the actions (enactment recall). Participants started from the first span with one-action instruction and progressed to the next length if four trials were correctly recalled at a given sequence length.

### Experiment 2

#### Participants

Thirty-six native Mandarin Chinese speakers were recruited through phone appointment. There were 20 females and 16 males, aged from 19 to 28, with a mean age of 23.11 and 16.43 mean years of education. None of the participants attended the previous experiment.

#### Materials

The materials from Experiment 1 were used again, with the dual instructions condition combining video clips of demonstrated actions with simultaneous audio instructions.

#### Design and procedure

In a 3 × 2 mixed design, input modality was a within-subject variable, including spoken, demonstration, and dual (demonstration and spoken) conditions. Output modality was a between-subject variable, including verbal and enactment recall. The dependent variables were the same as those in Experiment 1. Each participant was first introduced to the experiment and then signed the consent form. As in Experiment 1, the participants were randomly assigned to either the verbal or enacted recall condition, and each participant completed three conditions, i.e., spoken, demonstration and dual condition. The procedures in the spoken and demonstration condition were the same as those in Experiment 1. In the dual condition, participants watched demonstrated actions on a computer screen on a display in front of them while listening to the corresponding spoken instructions at the same time.

## Additional Information

**How to cite this article**: Yang, T.-x. *et al.* The influence of input and output modality on following instructions in working memory. *Sci. Rep.* **5**, 17657; doi: 10.1038/srep17657 (2015).

## Supplementary Material

Supplementary Information
