# Supplementary material for: The influence of input and output modality on following instructions in working memory
Source: Sci Rep. 2015 Dec 4;5:17657. doi: 10.1038/srep17657 (PMC4669483; doi:10.1038/srep17657)
Supplement: Supplementary Information [file srep17657-s1.pdf]

Supplementary materials

Title: The influence of input and output modality on following instructions in working memory

Authors: Tian-xiao Yang[1*], Richard J. Allen[2], Qi-jing Yu[3], Raymond C.K. Chan[1]

Supplementary material 1

Examples of instructions from one of the instructional lists

Level 1 involves instructions containing one action, such as 'Pull the yellow ruler'

Level 2 'Pick up the blue pencil then put it into the white basket'

Level 3 'Pick up the white rubber then put it into the yellow basket, and push the red pencil'

Level 4 'Push the red bag, and spin the black pencil, and pick up the blue ruler then put it into the green folder'

Level 5 'Pick up the blue ruler then put it into the yellow basket, and spin the red pencil and push the black bag and touch the green folder'

Level 6 'Spin the green rubber and pull the black bag, and touch the white basket then push the blue ruler and push the white basket and pull the black bag'

Supplementary Material 2

Passing of a span length was defined as correct recall of four out of six instructional sentences at that level of span. Each correctly recalled instruction sentence was assigned a score of 0.25. Therefore, the span score achieved by an individual was the number of correctly recalled instruction sentences multiplied by 0.25, ranged from 0 to 6.

Table S1

Means (standard errors) of span in two experiments

	Verbal recall	Enacted recall	Total
Experiment 1			
Spoken	3.72 (0.13)	4.31 (0.14)	4.01 (0.10)
Written	3.92 (0.15)	4.08 (0.11)	4.00 (0.09)
Demonstration	4.65 (0.18)	4.76 (0.12)	4.71 (0.11)
Mean	4.19 (0.11)	4.38 (0.07)	4.24 (0.07)
Experiment 2			
Spoken	3.85 (0.19)	4.47 (0.12)	4.16 (0.12)
Demonstration	4.19 (0.17)	4.60 (0.17)	4.40 (0.12)
Dual	4.06 (0.17)	4.68 (0.14)	4.37 (0.12)
Mean	4.03 (0.13)	4.58 (0.11)	4.31 (0.10)

Note. Dual condition involved simultaneous representation of spoken and demonstration.
